# Environmental gradients shape microbial community structure and ecosystem processes in Antarctic lakes on King George Island

**DOI:** 10.1038/s41598-025-21587-1

**Published:** 2025-10-27

**Authors:** Jiyoung Yoon, Hanbyul Lee, Yeongcheol Han, Sun-Yong Ha, Min Kyung Lee, Kitae Park, Hyejin Jung, Cheon Yun Kang, Yong-Un Chae, Jang-Cheon Cho, Ok-Sun Kim

**Affiliations:** 1 Division of Life Sciences, Korea Polar Research Institute, 26 Songdomirae-ro, Yeonsu-gu, Incheon, 21990 Republic of Korea; 2 Department of Biological Sciences and Bioengineering, Inha University, 40 Soseong-ro, Michuhol-gu, Incheon, 22201 Republic of Korea; 3 Division of Glacier & Earth Sciences, Korea Polar Research Institute, 26 Songdomirae-ro, Yeonsu-gu, Incheon, 21990 Republic of Korea; 4 Division of Marine Sciences, Korea Polar Research Institute, 26 Songdomirae-ro, Yeonsu- gu, Incheon, 21990 Republic of Korea; 5 Department of Environmental Sciences and Biotechnology, Hallym University, 1, Hallymdaehak-gil, Chuncheon, 24252 Republic of Korea; 6 Department of Geological Sciences, Pusan National University, 2, Busandaehak-ro 63beon- gil, Geumjeong-gu, Busan, 46241 Republic of Korea; 7 Institute of Environmental Geosciences, Pukyong National University, 45 Yongso-ro, Nam- gu, Busan, 48513 Republic of Korea

**Keywords:** Microbial diversity, Antarctic lakes, Oligotrophic, Amplicon sequencing, Extreme environment, Biogeochemistry, Ecology, Ecology, Environmental sciences, Microbiology

## Abstract

Antarctic lakes are extreme, oligotrophic habitats that contain microbial communities distinct from those of temperate freshwater systems. Our central question was whether these lakes host microbial communities distinct from those of non-Antarctic freshwater systems, and how environmental variability drives community differences among Antarctic lakes themselves. We analyzed the microbial community across five lakes on King George Island via high-throughput sequencing of amplicon sequence variants (ASVs) and biogeochemical profiling. The microbial communities were dominated by Bacteroidota, Actinomycetota, and Pseudomonadota, but varied strongly with environmental gradients such as salinity, sulfate, methane, and organic carbon. Hybrid ASVs, which were ubiquitous in both water and sediment, comprised the majority of sequences and indicate that dispersal processes, alongside environmental filtering, jointly structure lake microbial communities. Functional predictions further revealed lake- and habitat-specific pathways for carbon, nitrogen, and sulfur cycling, linking microbial diversity to ecosystem processes. These findings highlight how Antarctic lake microbes are shaped by both local selective pressures and cross-habitat exchange, providing critical insights into the resilience and vulnerability of polar freshwater ecosystems under climate change.

**Supplementary Information:**

The online version contains supplementary material available at 10.1038/s41598-025-21587-1.

## Introduction

 Antarctica, known as the coldest and driest continent, is covered by extensive ice sheets, with ice-free regions, both permanent and seasonal, accounting for only approximately 2% of the continent’s surface^1^. These limited ice-free areas host ecosystems characterized by persistent subzero temperatures, intense ultraviolet (UV) radiation, and frequent freeze-thaw cycles^2,3^. These conditions restrict biodiversity, resulting in ecosystems dominated by highly adapted microbial communities with simplified food webs^4,5^. The microorganisms inhabiting Antarctic ecosystems exhibit distinctive biochemical, physiological, and genetic adaptations, offering important understanding into adaptive mechanisms under extreme environmental conditions^5,6^. Among these ecosystems, Antarctic lakes are characterized by diverse ecological niches differing in size, depth, and biogeochemistry. These lakes are particularly sensitive indicators of warming as glacial meltwater inputs modify their hydrology, chemistry, and biology, potentially leading to lake expansion or formation of new freshwater habitats^7,8^.

Recent studies of Antarctic lakes have revealed that, even under extreme nutrient scarcity, microbial communities harbor a broad functional capacity to drive biogeochemical cycles^9,10^. For example, depth-resolved metagenomes from Lake Bonney showed that cyanobacteria fix CO₂in the oxygenated surface layer, while the dark, suboxic deep waters are dominated by organotrophs and chemolithotrophs that generate energy using nitrogen and sulfur species along the lake’s redox gradient^9^. In Lake Hazen (High Arctic), genome-resolved metagenomics uncovered numerous cold-adapted microbial lineages with enriched lipid metabolism and streamlined nutrient transporter profiles^10^. These studies have offered essential information on microbial biodiversity and functional roles; however, applying their findings to broader Antarctic regions remains challenging due to the high environmental heterogeneity^11,12^.

King George Island, part of the Maritime Antarctic region, hosts numerous lakes exhibiting a broad range of environmental characteristics influenced by proximity to the ocean, terrestrial inputs, glacial meltwater contributions, and underlying geology^13–16^. Although a few microbial studies have been conducted on Fildes Peninsula lakes, research addressing comprehensive microbial community structures and their environmental determinants across different lakes on King George Island is notably lacking^13,17,18^. Sediments are major reservoirs of organic matter, nutrients, and microbial diversity, influencing lake chemistry and microbial dynamics under nutrient-limited Antarctic conditions^2,19^. In particular, sediment-associated microbial communities, which are key players in biogeochemical cycling and ecosystem functioning in Antarctic lakes, have received relatively little attention despite their ecological importance^20,21^. Therefore, understanding the microbial community structure within both water columns and sediments is essential for elucidating the comprehensive ecological dynamics of Antarctic-lake systems.

Importantly, most previous studies of Antarctic lakes have focused on either water columns or sediments separately, leaving a critical gap in understanding how microbial communities are structured and function across these connected habitats. Comparing microbial communities between water and sediments is essential for revealing cross-habitat linkages, such as microbial exchange at the sediment-water interface, habitat-specific adaptations, and differential contributions to biogeochemical cycling. This perspective is especially important in Antarctic lakes, where strong environmental gradients and extreme oligotrophy magnify the ecological significance of microbial processes.

The aim of this study was to investigate the composition, structure, and ecological roles of microbial communities inhabiting water columns and sediments from five lakes on King George Island, Antarctica. These lakes vary considerably in their physicochemical properties, providing ideal model systems to examine how environmental gradients shape microbial communities in extreme habitats. Specifically, we address the following research questions:

1. Do microbial communities in Antarctic lakes resemble those of non-polar oligotrophic lakes with comparable trophic status, or do they exhibit unique taxonomic and ecological traits?
2. Do small environmental differences among nearby Antarctic lakes lead to major changes in microbial community composition and function?
3. Are microbial assemblages in Antarctic lakes structured more strongly by dispersal processes linking water and sediments, or by environmental filtering imposed by lake-specific physicochemical conditions?

By answering these questions, we aim to advance the understanding of microbial community diversity and biogeochemical functions in extreme Antarctic freshwater environments. Our results reveal strong associations between microbial distributions and environmental gradients such as salinity, sulfate, and organic carbon, highlighting how shifts in these factors under ongoing climate change may substantially alter microbial biodiversity and ecosystem functions in Antarctic lakes.

## Materials and methods

### Site description

**Fig. 1:**
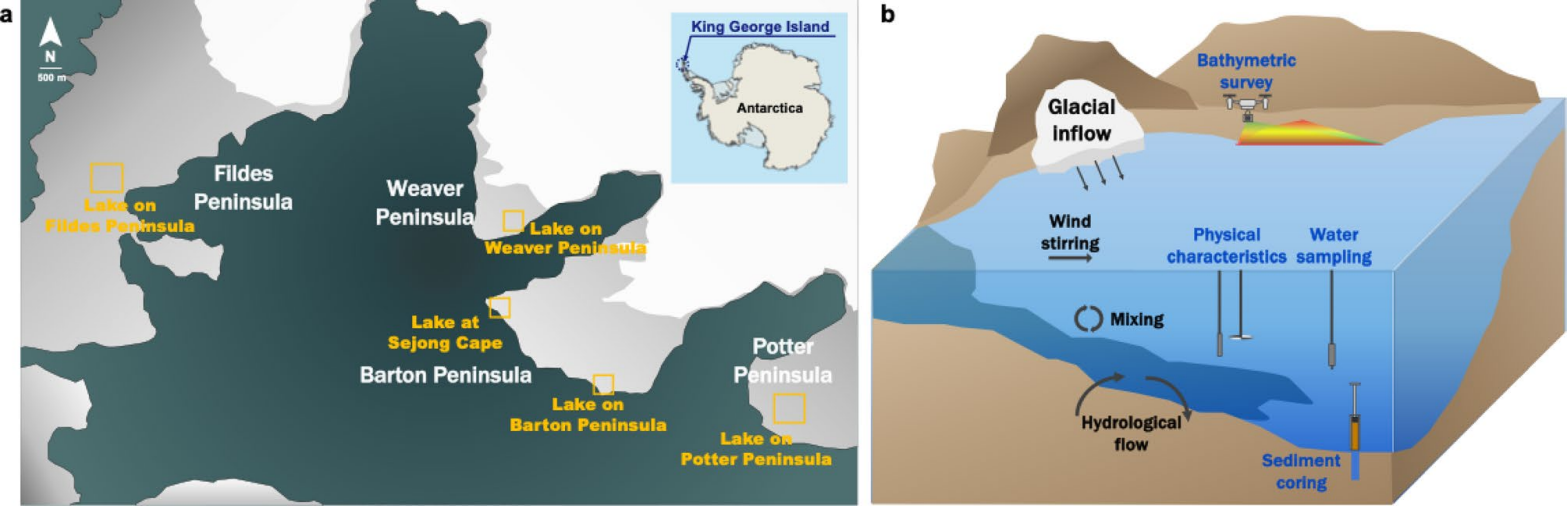
Location and schematic structure of the lakes investigated in this study. (**a**) Map showing sampling sites across five lakes located on four peninsulas of King George Island, Antarctica. (**b**) Schematic illustration of lake structures and sampling strategy employed during fieldwork.

The five lakes included in the study are located on King George Island, Antarctica (Fig. 1; Table 1). King George Island (61°50′S to 62°15′S; 57°30′W to 59°01′W) is the largest island in the South Shetland Islands archipelago. Most of its surface is covered by glaciers, and ice-free terrain is exposed only along the shorelines in restricted areas. Due to the strong maritime influence, the climate is relatively humid and mild compared with that of the Antarctic Peninsula^1^.

**Table 1:** Major geographical and environmental characteristics of each lake in the Antarctic region.

Lakes	Elevation	Area	Max depth	Distance to shoreline	Distance to glacier	Distance to station
Lake on Barton (LB)	30.0 m	2,714.0 m^2^	2.0 m	248.0 m	1,898.0 m	2,882.0 m
Lake at Sejong Cape (LS)	2.0 m	12,180.0 m^2^	1.5 m	130.0 m	3,035.0 m	417.0 m
Lake on Fildes (LF)	15.0 m	81,638.0 m^2^	15.0 m	473.0 m	3,381.0 m	600.0 m
Lake on Potter (LP)	45.0 m	79,018.0 m^2^	6.5 m	854.0 m	1,669.0 m	1,053.0 m
Lake on Weaver (LW)	100.0 m	3,243.0 m^2^	2.0 m	519.0 m	850.0 m	1,882.0 m

The Fildes Peninsula is the largest ice-free area on King George Island and is one of the most active regions in Antarctica in terms of human activity, featuring numerous lakes and six research stations. The Weaver Peninsula and Barton Peninsula are located in the southwestern part of King George Island and are separated by Marian Cove. The ice-free areas of the Barton Peninsula are characterized by gently sloping terrain, including a wide central plain at an elevation of 90–180 m^22^. The Barton Peninsula has relatively abundant vegetation, and lake on Barton Peninsula (LB) is particularly vegetated^1^. The Potter Peninsula’s terrain is characterized by glacial features with cliffs along the coast and somewhat smooth, hilly inland areas. The climate is primarily cool and maritime, typical of ice-free Antarctic coastal regions covered by lower plants such as mosses and lichens^23^.

### Sample collection and physicochemical properties

For one month, the microbial community in five lakes located on King George Island was monitored. Water samples for bacterial diversity and physicochemical analyses were collected from five lakes on King George Island in January 2023. Surface and deeper water samples were collected using a portable peristaltic pump equipped with new tubing at each site. Prior to collection, the tubing was flushed with lake water for more than one minute to minimize potential contamination. As no clear stratification was observed during fieldwork, samples were taken from upper and lower portions of the water column, representing conditions near the surface and bottom. In the deepest lake (lake on Fildes Peninsula, LF), three depths were sampled to better capture vertical variability in physicochemical conditions. For physicochemical property analysis, samples were directly filtered through sterile 0.2 μm capsule filters. Prior to sample collection, vertical depth profiles of temperature, conductivity, and dissolved oxygen were measured using a YSI multiparameter instrument (YSI Inc., USA). To analyze the diversity of the bacterial community, water samples were filtered through 0.2 μm (Whatman, USA) membrane filters. Filtered samples were stored at -20 °C during shipment and then at -80 °C until analysis. The concentrations of inorganic nutrients (NO_2_^−^+ NO_3_^−^, NH_4_^+^, PO_4_³⁻) were measured using a 4-channel continuous flow analyzer (QuAAtro, SEAL Analytical, UK)^24^. Dissolved organic carbon (DOC) was determined using a Shimadzu TOC-V series total organic carbon analyzer in acidified samples filtered through GF/F filters^25^. For the analysis of total chlorophyll a (Chl-a), high-performance liquid chromatography (HPLC) was used. Water samples were filtered using Whatman GF/F filters, and Chl-a was extracted overnight in 90% acetone. The concentration of Chl-a was then measured using HPLC^26^. Sulfate (SO_4_^2−^) concentrations were measured using ion chromatography (Dionex Integrion HPIC, Thermo Fisher). Dissolved methane (CH_4_) and nitrous oxide (N_2_O) in water samples were extracted using the headspace method, in which dissolved gases were equilibrated between water and gas phases by shaking. The equilibrated gas was then analyzed using a cavity ring-down spectrometer (CRDS), and the measured headspace concentrations were converted to dissolved CH_4_ and N_2_O concentrations^27,28^.

Sediment cores were collected using a piston corer with a polycarbonate liner, sealed immediately, and transported to the laboratory within one hour of collection. In the laboratory, the sediment cores were aseptically subsampled at 3 cm intervals using sterile stainless steel blades. The resulting subsamples were subsequently stored under sterile conditions at -80 °C for downstream molecular (DNA extraction) and sediment geochemical analyses.

All geochemical proxies for sediment cores were measured at 1 cm intervals at the Korea Polar Research Institute (KOPRI). The water content of each subsample was determined by measuring the difference between the wet and dry weights before and after freeze-drying at -80 °C. The total carbon (TC) and total nitrogen (TN) concentrations were measured by an organic elemental analyzer (FLASH 2000 NC Analyzer) with an analytical precision of less than ± 0.1%. The total inorganic carbon (TIC) concentration was measured using a UIC CO_2_ coulometer (Model CM5240). The calcium carbonate (CaCO_3_) concentration was calculated by multiplying the TIC concentration by 8.333^29^. The relative standard deviation for the CaCO_3_ concentration was ± 1%. The TOC concentration was calculated as the difference between TC and TIC. C/N ratios were calculated as TOC/TN. The biogenic silica concentration was measured using a continuous flow analyzer (SKALAR SANplus Analyzer) with a wet-alkaline extraction method modified from DeMaster^30^following the method of Kim et al^31^.. The biogenic opal concentration was calculated by multiplying the biogenic silica concentration by 2.4^32^. The relative error of the biogenic silica concentration in sediment samples was < 1%.

### DNA extraction

Water samples were filtered through filter paper (0.22 μm, Millipore) for DNA extraction. DNA was extracted using a FastDNA Spin Kit for Soil (MP Biomedicals, USA) according to the manufacturer’s instructions. The total DNA of the sediment samples was extracted from 0.5 g of sediment using a Power Soil DNA Isolation Kit (MoBio, USA) following the manufacturer’s instructions. The quality and concentration of gDNA were examined by standard electrophoresis.

### Amplification and illumina miseq sequencing

DNA libraries were constructed using the Illumina MiSeq platform with 16 S rRNA gene amplicons. The extracted DNA was amplified with the primer set 341 F (5′ CCTACG GGNGGCWGCAG 3′) and 805R (5′ GACTACHVGGGTATCTAATCC 3′), which is specific to the V3-V4 region of the 16 S rRNA gene, and the Illumina overhang adaptor was incorporated according to the manufacturer’s instructions. The thermocycler settings for amplification of the 16 S rRNA gene were as follows: initial denaturation at 95 °C for 3 min; 25 cycles of 95 °C for 30 s, 55 °C for 30 s, and 72 °C for 30 s; and final elongation at 72 °C for 5 min. A multiplexed amplicon library was generated and then sequenced using a MiSeq platform (Illumina) to obtain 2 × 300 bp paired end reads (Macrogen Inc., Korea).

### Bioinformatics analyses of 16 S rRNA gene sequences

To enable comparison with temperate lakes, we incorporated amplicon data from three oligotrophic lakes previously reported in the literature (Table S1) into subsequent analyses. The sequence data were processed and analyzed using QIIME2-2023.2^33^. The imported data were trimmed with cutadapt using the primer sequences and then processed with DADA2 for quality control and denoising^34^. Taxonomic classification of amplicon sequence variants (ASVs) was performed using the feature classifier classify-sklearn based on SILVA 138 at 99% sequence similarity^35^. The data were then filtered to remove mitochondrial and chloroplast reads. Rarefaction curves for evaluating sufficient sequence depth were created on the basis of alpha diversity with a depth of 20,000 (Fig. S1).

The microbial communities across water and sediment samples from the Antarctic lakes were classified into three broad groups based on ASVs: (1) hybrid ASVs present in both water and sediment samples; (2) water-specific ASVs found exclusively in water; and (3) sediment-specific ASVs found exclusively in sediments. Each of these categories was further classified on the basis of their occurrence pattern as core (detected across all sites), location-specific (restricted to a single site), or other (neither core nor site-specific).

The PICRUSt2 plugin for QIIME2 (q2-picrust2 ver. 2023.2)^36^ was employed to predict functional abundances on the basis of 16 S rRNA gene sequencing data. The functional composition of the data was predicted based on the KEGG Orthology (KO) database^37^ using PICRUSt2. KO terms were filtered to retain only those associated with major biogeochemical cycling in the lakes, following Lee et al.^9^. KO abundances were then analyzed using hierarchical clustering of columns (samples) based on Pearson correlation distance, and visualized as a heatmap with the pheatmap package in R (v4.3.1).

### Statistical analysis

Diversity metrics were calculated with diversity core-metrics-phylogenetics, with sequences rarefied to 20,000. The community structure of all the samples was visualized using nonmetric multidimensional scaling (NMDS) based on Bray-Curtis dissimilarity using the vegan 2.6-4 package^38^ in R. The vectors representing environmental variables were calculated with the envfit function in R and used in NMDS. Environmental fitting (envfit) was performed with 999 permutations to assess correlations between microbial community ordinations and environmental variables. Reported p-values are uncorrected, as our analysis focused on identifying the most influential environmental drivers rather than testing a large number of independent hypotheses. Differences in microbial community composition between the lakes were assessed through multiple permutational analysis of variance (PERMANOVA) using the adonis function (permutations = 999, alpha < 0.05). Indicator species analysis (ISA) was conducted to identify ASVs significantly associated with Antarctic lakes using the function indval in the “labdsv” package^39^. P-values were not adjusted for multiple testing, and results are interpreted with caution given the exploratory nature of this analysis. To analyze differences in alpha diversity, a permutation test was performed^40^.

## Results

### Biogeochemical characteristics of Antarctic lakes

The biogeophysical characteristics of water samples from the selected five lakes on King George Island are summarized in Table 2. The mean water temperature during the austral summer was recorded at 5.8 °C, uniform across depths, indicating an absence of thermal stratification. The conductivity levels ranged between 95 and 130 µS/cm, while lake at Sejong Cape (LS), located closer to the coast, exhibited levels above 7000 µS/cm. The measured Chl-*a* concentrations were between 0.2 and 1.0 µg/L. The dissolved organic carbon (DOC) concentrations ranged from 10 to 30 µM. LB and LS had methane concentrations exceeding 70 nM. Sulfate concentrations displayed a wide range from 3 to 24 ppm, with the LS concentration exceeding 720 ppm. The average cell concentrations across the lakes ranged from 10^5^ to 10^6^ cells/ml, although lake on Weaver Peninsula (LW) exhibited significantly lower concentrations, at approximately 10^4^ cells/ml. These results indicate that, while Antarctic lakes are typically oligotrophic^41^, LB and LS exhibit relatively nutrient-rich conditions in comparison with the other lakes.

**Table 2:** Physicochemical and biological characteristics of each lake at the indicated sampling depths.

Environmental variables	LB		LS		LF			LP		LW	
	1 m	2.25 m	0.2 m	0.6 m	1 m	5 m	9 m	1 m	4.1 m	1 m	2.25 m
Temp. (°C)	5.9	5.9	6.2	6.3	6.3	6.3	6.3	5.8	5.8	5.6	5.5
Conductivity (µS/cm)	95.3	84.3	7168.0	7953.0	128.9	129.0	129.1	61.1	61.1	55.0	55.1
Chlorophyll *a* (µg/L)	0.5	1.1	0.6	0.6	0.3	0.2	0.3	0.5	0.6	0.2	0.2
Total nitrogen (µM)	9.0	6.0	13.0	3.0	2.0	1.0	1.0	2.0	1.0	1.0	1.0
Nitrate + nitrite (µM NO_3_^−^+NO_2_^−^)	2.8	4.2	0.8	0.7	0.9	1.3	1.7	1.1	1.0	1.2	1.0
Ammonium (µM NH_4_^+^)	1.4	1.9	1.1	1.2	1.7	3.0	3.2	1.9	1.8	1.1	1.1
C/N	68.6	9.8	6.2	50.5	10.2	17.8	8.4	17.8	31.2	10.4	13.2
Dissolved organic carbon (µM)	625.7	539.6	83.0	152.0	21.4	13.1	12.1	32.6	26.8	12.1	14.4
Sulfate (ppm SO_4_^2−^)	23.5	23.4	724.1	721.3	16.1	15.9	15.9	3.1	3.1	3.8	4.0
Phosphate (µM PO_4_^3−^)	1.6	1.7	0.1	0.0	0.1	0.1	0.1	0.4	0.4	0.0	0.0
Nitrous oxide (nM N_2_O)	20.8	20.5	20.4	20.3	15.4	14.6	14.7	13.7	14.2	15.3	13.9
Methane (nM CH_4_)	77.8	80.4	80.7	80.6	5.9	4.9	4.3	7.4	8.2	6.2	4.2
Cell concentration (cells/mL)	1.4 × 10^6^	1.0 × 10^6^	5.4 × 10^5^	5.4 × 10^5^	5.0 × 10^5^	1.0 × 10^5^	7.5 × 10^4^	8.7 × 10^5^	9.2 × 10^5^	3.2 × 10^4^	1.3 × 10^5^

**Table 3:** Physicochemical and biological characteristics of the sediment samples.

	LB								LS				LF			LP									
	0–3 cm	3–6 cm	6–9 cm	9–12 cm	12–15 cm	15–18 cm	18–21 cm	21–24 cm	0–3 cm	3–6 cm	6–9 cm	9–12 cm	0–3 cm	3–6 cm	6–9 cm	0–3 cm	3–6 cm	6–9 cm	9–12 cm	12–15 cm	15–18 cm	18–21 cm	21–24 cm	24–27 cm	27–30 cm
TC^a^	4.98	2.87	3.2	4.13	3.11	1.29	0.78	0.8	10.6	9.25	8.91	8.72	0.27	0.22	0.25	0.2	0.08	0.05	0.05	0.03	0.03	0.03	0.03	0.05	0.04
TOC^b^	4.93	2.85	3.17	4.06	3.05	1.26	0.77	0.78	10.58	9.22	8.89	8.7	0.27	0.22	0.25	0.2	0.08	0.05	0.05	0.03	0.03	0.03	0.03	0.05	0.04
TIC^c^	0.05	0.02	0.03	0.07	0.06	0.02	0.01	0.02	0.02	0.03	0.02	0.02	0	0	0	0	0	0	0	0	0	0	0	0	0
TN^d^	0.82	0.44	0.51	0.65	0.55	0.24	0.15	0.15	1.42	1.33	1.15	1.18	0.02	0.02	0.02	0.02	0.01	0	0	0	0	0	0	0	0
Opal	0.39	0.19	0.26	0.59	0.46	0.21	0.12	0.16	0.17	0.22	0.19	0.16	0	0	0	0.01	0	0	0	0	0	0.01	0	0	0.01
Water content (%)	59.1	57.26	61.56	36.59	30.74	27.15	27.3	26.93	81.13	81.59	79.25	78.36	35.26	40.87	40.69	38.61	32.81	29.42	28.17	25.42	25.08	26.3	29.73	24.39	28.06

^a^Total carbon.

^b^Total organic carbon.

^c^Total inorganic carbon.

^d^ Total nitrogen.

LS sediment had the highest TC concentrations (8.72% to 10.6%), indicating organic-rich conditions (Table 3). In contrast, LF displayed notably lower TC values between 0.22% and 0.27%, suggesting limited organic matter accumulation. LB and lake on Potter Peninsula (LP) showed intermediate TC concentrations, with the values for LB ranging from 0.78% to 4.98%, while LP presented significantly lower values, between 0.03% and 0.20%. Total organic carbon (TOC) mirrored the patterns observed for TC, reinforcing the dominant contribution of organic carbon to TC. LS sediments were particularly rich in TOC (8.70-10.58%), whereas LF and LP had much lower values (0.22–0.27% and 0.03–0.20%, respectively). Similarly, LB demonstrated moderate levels of TOC (0.78–4.93%). Inorganic carbon (TIC) was consistently low across all sediments, rarely exceeding 0.07%, indicating minimal inorganic carbon contributions. The total nitrogen (TN) concentration exhibited spatial patterns analogous to those of carbon. LS presented the highest nitrogen levels (1.15–1.42%), followed by LB, with intermediate values (0.15–0.82%), and LF and LP presented the lowest nitrogen content (< 0.02%). The opal content, indicative of the presence of biogenic silica, was highest in LB sediments, particularly at depths of 9–12 cm (0.59%), indicating variations in biogenic sedimentation processes. LS had intermediate opal concentrations (0.16–0.22%), while LP and LF generally lacked detectable opal, indicating lower biogenic silica production or preservation. The water content of the sediments varied significantly, with the highest values observed in LS (78.36–81.59%), suggesting that the sediment was characterized by high porosity and water saturation. Sediment from LB exhibited moderate water contents ranging from 26.93% to 61.56%, with the upper layers showing higher moisture. LF and LP had notably lower and more consistent water contents, generally below 40%, which decreased with depth. These results highlight substantial spatial variability in sediment biogeochemistry among Antarctic lakes, with LS and LB sediments characterized by higher nutrient and organic matter contents than LF and LP. These differences reflect the occurrence of distinct sedimentary and ecological processes within these lakes.

### Microbial community composition across lakes and depths

In the bacterial community analysis of the five Antarctic lakes, Bacteroidota (43%) was identified as the most abundant phylum, followed by Actinomycetota (24%) and Pseudomonadota (19%) (Fig. S2). Since the lakes were not stratified at the time of sampling, most depths within each lake exhibited a homogeneous distribution. In the relatively nutrient-rich lakes LB and LS, Bacteroidota was dominant (78% and 70%, respectively). Actinomycetota was observed at a very low proportion in LS (1%), a brackish lake located close to the shoreline, while it was predominant in LW (49%) and LF (37%). In LF and LP, Verrucomicrobiota accounted for a high proportion (24% and 12%, respectively). Unlike the other lakes, LW exhibited considerable differences in microbial community composition across depths. In the surface layer of LW, Actinomycetota (60%) was dominant, followed by Bacteroidota (19%), whereas in the deeper layer, Pseudomonadota (60%) and Actinomycetota (38%) were dominant.

At the genus level, *Flavobacterium* (Bacteroidota) dominated LB (57%), LS (62%), and LP (37%). In LF, Sporichtyaceae and the hgcl clade, which are associated primarily with freshwater environments, were detected, and uncultured Verrucomicrobiae were observed only in this lake. *Polaromonas* was found at all sites except LS, while *Pseudorhodobacter* was identified in the deeper layers of LS. In LW, genera such as *Sphingomonas*, *Rhodococcus*, and *Brevundimonas* were identified, none of which were observed in other lakes.

Sediment microbial compositions differed notably from those in water samples (Fig. S2). Pseudomonadota, Bacteroidota, and Bacillota were consistently abundant, but their relative contributions varied across lakes. LS sediments were dominated by Bacteroidota (~ 40–45%) and contained notable proportions of sulfate-reducing Desulfobacterota (~ 10%), reflecting sulfate-rich conditions. LF sediments contrasted by harboring high Actinomycetota (~ 30–40%), while LB showed mixed dominance of Pseudomonadota and Bacillota, with moderate contributions from Actinomycetota. LP sediments were more heterogeneous across depths, with fluctuating proportions of Pseudomonadota (20–40%) and Verrucomicrobiota. These differences indicate lake-specific environmental drivers shaping distinct sedimentary microbial assemblages.

### Distribution patterns of habitat-specific microbial ASVs in Antarctic lake environments

**Fig. 2:**
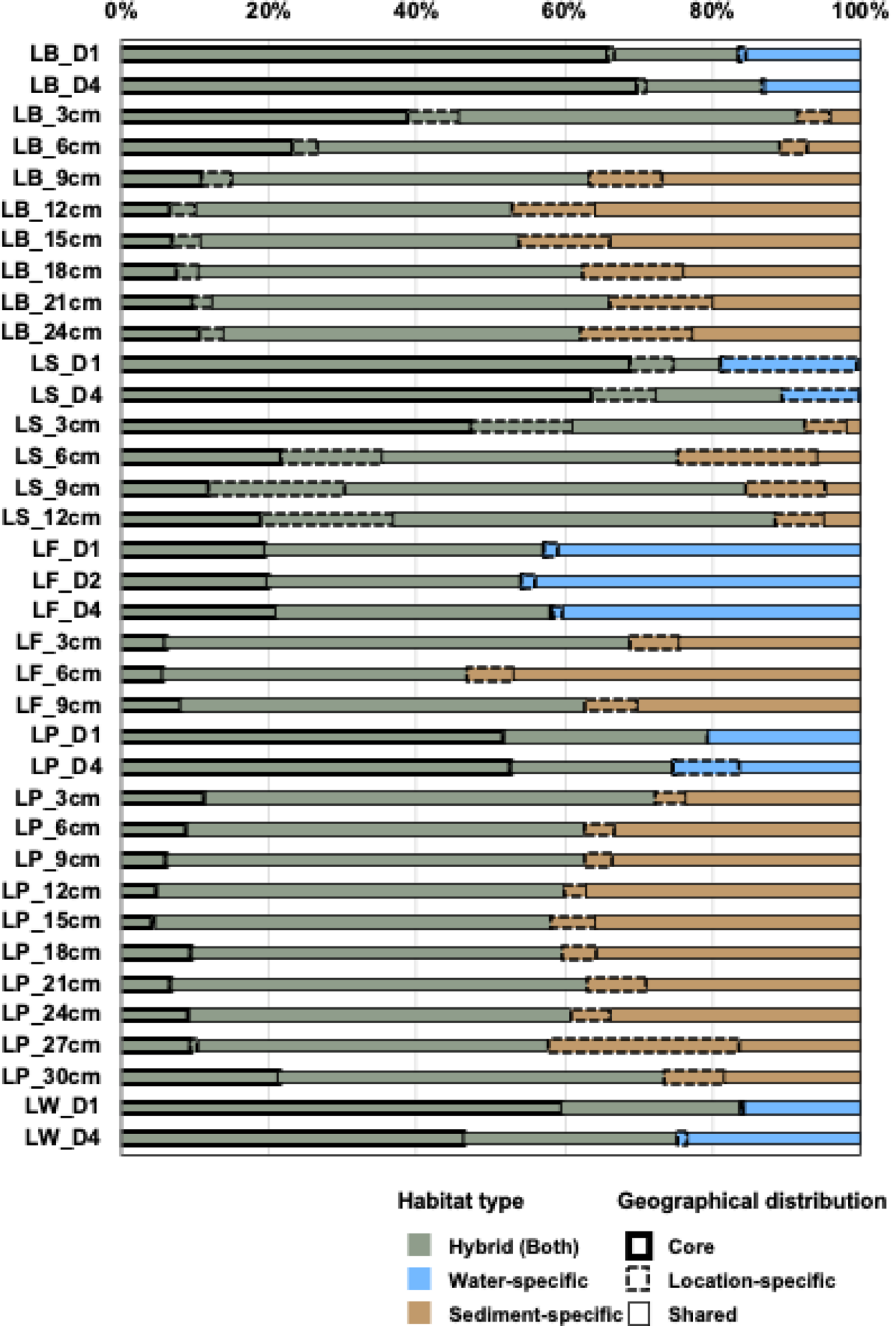
Distribution patterns of habitat-specific microbial ASVs in Antarctic lake environments. ASVs were classified as hybrid (present in both water and sediment), water-specific, or sediment-specific. Each group was further categorized by spatial distribution: core (found at all sites), location-specific (found at a single site), or other (neither core nor location-specific).

Overall, hybrid ASVs (i.e., those present in both water and sediment samples) dominated the microbial communities, accounting for approximately 70% of the total sequences across all samples (Fig. 2). This distribution pattern reflects ecological mechanisms such as dispersal across habitats and broad environmental tolerance, which may facilitate the persistence of hybrid ASVs under diverse lake conditions. Specifically, of the total 3,134 ASVs detected, hybrid ASVs comprised 1,057, whereas water-specific and sediment-specific ASVs comprised 437 and 1,640, respectively, indicating a higher relative abundance of hybrid ASVs despite their lower diversity. In water samples, water-specific ASVs represented approximately 25% of sequences, whereas in sediment samples, sediment-specific ASVs represented approximately 32%. Although the relative abundance of hybrid ASVs appeared higher in water samples (75%) than in sediment samples (68%), statistical analysis showed that this difference was not significant (*p* > 0.05). The prevalence of hybrid ASVs suggests that dispersal between water and sediments and tolerance to a wide range of conditions may facilitate their persistence across habitats. Core ASVs, which were found consistently across all sites, predominantly belonged to the hybrid category, further indicating broad ecological flexibility. By contrast, habitat-specific ASVs revealed distinct patterns: sediment-specific ASVs were generally more abundant than water-specific ASVs, which may be partly explained by environmental filtering, given that sediments provide heterogeneous microenvironments that select for a wider range of specialized taxa. Conversely, in LF, water-specific ASVs exhibited unusually high dominance, potentially due to the lake’s greater depth (maximum 15 m) and reduced sediment or terrestrial inputs. Interestingly, water-specific ASVs in LF showed a particularly low proportion of location-specific ASVs, likely due to the larger size of the lake and consequent broad distribution of taxa. In contrast, LS water samples were unique among the lakes and were dominated primarily by water-specific, location-specific ASVs, suggesting that strong environmental filtering imposed by lake-specific physicochemical conditions.

### Community structure and environmental drivers forming Antarctic lake microbial communities

**Fig. 3:**
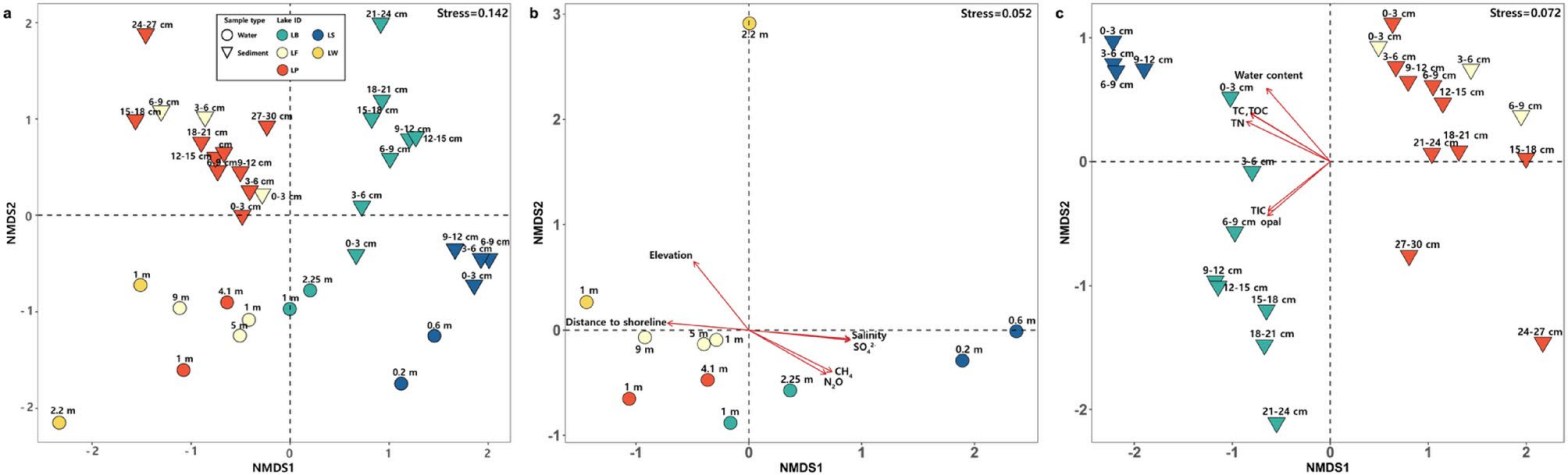
Microbial community structure and associated environmental drivers in Antarctic lakes. (**a**) Nonmetric multidimensional scaling (NMDS) ordination based on Bray-Curtis dissimilarity, illustrating microbial community composition of water (circles) and sediment (triangles) samples collected from five Antarctic lakes. (**b**) NMDS plot depicting microbial communities from water samples, with environmental factors (distance to shoreline, elevation, salinity, and concentrations of methane, nitrous oxide, and sulfate) identified as significant by envfit analysis represented by red arrows. (**c**) NMDS plot illustrating microbial communities from sediment samples, with significant environmental factors (concentrations of opal, TC, TIC, TN, TOC, and water content) indicated by red arrows. Environmental vectors shown were determined to be significantly correlated with microbial community structure (envfit analysis; *p* < 0.05).

NMDS based on Bray-Curtis dissimilarities clearly distinguished the microbial communities according to individual Antarctic lakes (Fig. 3(a)). LF and LP microbial assemblages clustered closely, indicating similar structures. In contrast, LS exhibited a distinct microbial composition and was markedly separated from the other lakes. This pattern of community differentiation was even more pronounced in sediment samples than in water samples. While water samples (excluding LS) clustered closely in the NMDS ordination space, sediment samples formed clearly distinct clusters corresponding to individual lakes. Furthermore, shallow sediment communities showed greater similarity to water-column communities, whereas greater sediment depth corresponded to greater divergence, amplifying differences in microbial composition among the lakes.

An environmental fitting (envfit) analysis on the NMDS ordinations revealed that salinity was the primary driver of microbial community composition in water samples, whereas nutrient-related factors were most influential in sediments. In water samples, methane (r² = 0.8, *p* < 0.001), sulfate (r² = 0.9, *p* < 0.001), and salinity (r² = 0.9, *p* < 0.01) demonstrated the strongest associations with microbial community structure, highlighting their roles in influencing microbial distributions (Fig. 3(b)). Additional significant variables included nitrous oxide (N_2_O, r² = 0.5, *p* < 0.01), elevation (r² = 0.7, *p* < 0.01), and distance from the shoreline (r² = 0.6, *p* < 0.01). Elevation primarily distinguished LW from LB and LS, whereas high salinity and sulfate concentrations drove the unique microbial composition of LS water samples. Sediment microbial communities were also significantly correlated by several environmental parameters, notably water content (r² = 0.8, *p* < 0.01), TC(r² = 0.9, *p* < 0.01), TOC (r² = 0.9, *p* < 0.01), TN (r² = 0.8, *p* < 0.01), TIC (r² = 0.6, *p* < 0.01), and opal concentration (r² = 0.6, *p* < 0.01) (Fig. 3(c)). Among these variables, water content, TC, TOC, and TN exhibited particularly high values in LS sediment samples, further highlighting the unique environmental conditions of LS.

### Predictive functional potential of microbial communities

**Fig. 4:**
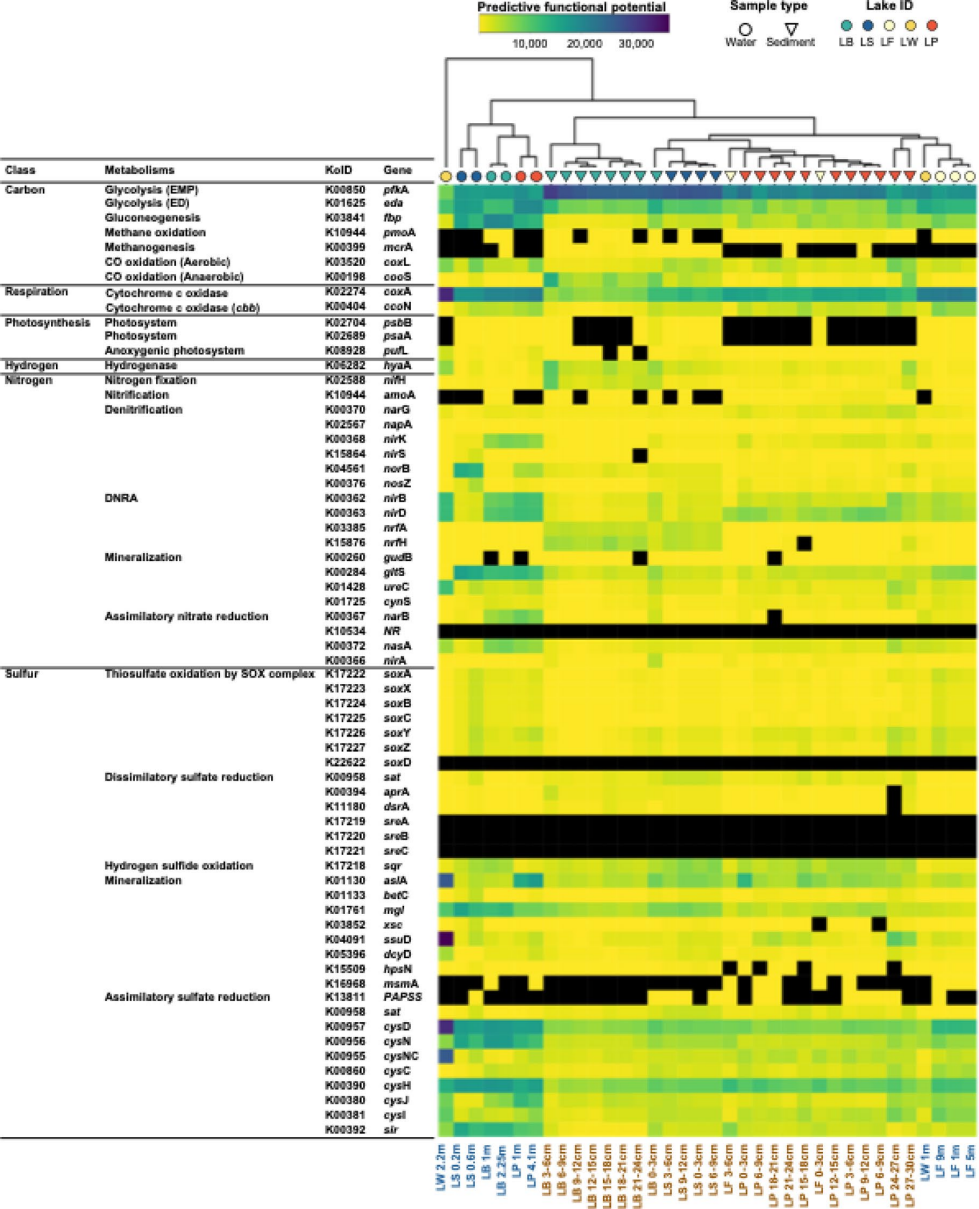
Predictive functional potential of microbial communities in Antarctic lake samples, inferred from 16 S rRNA gene amplicon data using PICRUSt2. Functional profiles were clustered by sample (columns) based on hierarchical clustering with Pearson correlation distance and visualized as a heatmap with the pheatmap package in R (v4.3.1). Sample types are indicated by color-coded labels: blue for water samples and brown for sediment samples. Symbols represent different sample categories. Abundance values are shown as a color gradient, with black indicating zero values.

To assess the contributions of microbial communities to biogeochemical cycling, we performed predictive functional profiling using PICRUSt2 based on the amplicon data (Fig. 4). The analysis revealed clear clustering patterns, with water and sediment samples forming distinct groups, and functional profiles further separating by lake within each habitat type. In carbohydrate metabolism, glycolysis via the EMP (Embden-Meyerhof-Parnas) pathway was significantly higher in sediments of LB and LS (on average 26,703; *p* < 0.05) than in other water and sediment samples (18,749). In contrast, glycolysis potential via the ED (Entner-Doudoroff) pathway was consistently greater in water samples (14,598; *p* < 0.05) compared with sediment samples (8,030). Gluconeogenesis showed the highest potential in LB water samples (mean 19,616), but was markedly low in LB sediments (1,893). In respiration, cytochrome c oxidase potential was higher in water samples (21,685; *p* < 0.05) compared with sediments (11,940). Nitrogen fixation potential was generally low across all samples (1,174), although relatively elevated values were observed in sediments of LB and LP (2,024). Nitrification potential was nearly absent, whereas denitrification and DNRA (Dissimilatory nitrate reduction to ammonium) showed moderate enrichment in several water samples. Notably, LB and LP exhibited higher potentials for both denitrification (1,034) and DNRA (2,892), while LS was characterized by enhanced denitrification potential (3,318). Furthermore, LS, LB, and LP water samples displayed higher potentials for mineralization and assimilatory nitrate reduction. For sulfur cycling, water samples consistently exhibited greater potentials for SOX-mediated sulfate oxidation, mineralization, and assimilatory sulfate reduction compared to sediments.

### Distinct microbial composition and indicator species of Antarctic lakes compared with global oligotrophic lakes

**Fig. 5:**
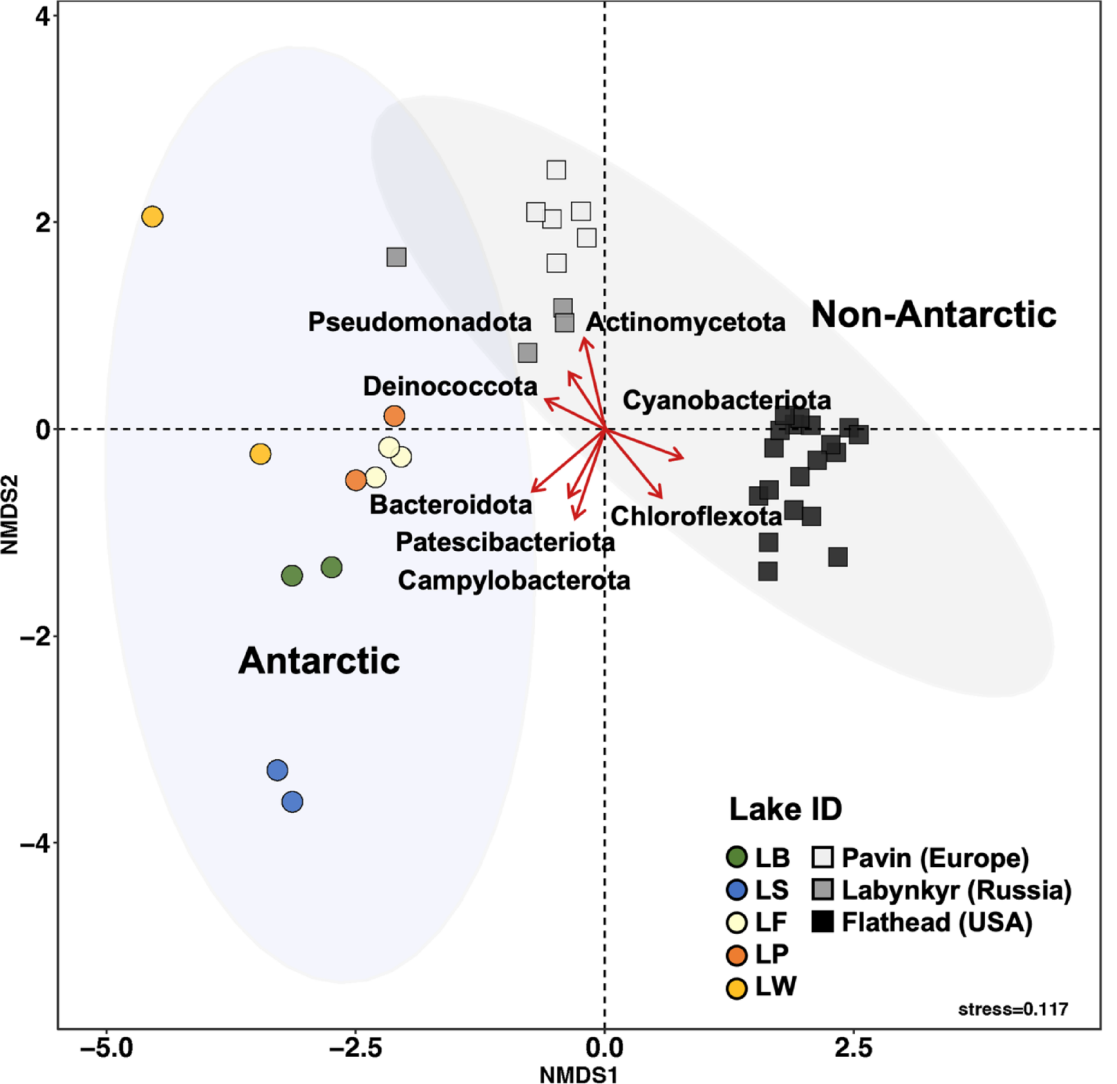
Bacterial communities in Antarctic and non-Antarctic lakes. Nonmetric multidimensional scaling (NMDS) ordination was generated on the basis of Bray-Curtis dissimilarity. Circular dots represent Antarctic lakes, while square dots represent non-Antarctic lakes. Red arrows indicate bacterial phyla significantly correlated with microbial community composition according to environmental fitting (envfit) analysis.

To investigate the differences in microbial composition between Antarctic lakes and oligotrophic lakes on other continents^42–44^, Bray-Curtis distance-based NMDS analysis was performed (Fig. 5). Antarctic lake microbial communities distinctly differed from that of non-Antarctic lakes (r² = 0.18, *p* < 0.001). Additionally, to identify which phyla are characteristic of Antarctic lakes, envfit analysis was conducted. The variations in microbial community composition can be significantly indicated by several specific bacterial phyla, such as Patescibacteriota (r² = 0.4, *p* < 0.001), Bacteroidota (r² = 0.5, *p* < 0.001), and Campylobacterota (r² = 0.5, *p* < 0.001), suggesting that each of these groups is associated with key environmental gradients microbial distribution patterns in the lakes. To identify taxa with significantly different compositions, indicator species analysis (ISA) was performed (Table 4). As a result, a total of 15 Antarctic and 4 non-Antarctic indicator ASVs were identified. Among the 15 Antarctic indicators, 7 ASVs belonged to Patescibacteriota, 4 to Pseudomonadota, 2 to Bacillota, and one each to Actinomycetota and Bacteroidota. In contrast, the non-Antarctic indicators included two ASVs from Bacteroidota and one ASV each from Actinomycetota and Pseudomonadota.

**Table 4:** Indicator ASVs for Antarctic and non-Antarctic lakes selected through ISA and their taxonomic information.

Group	IndVal	*p* value	ASV ID	Phylum	Genus	Closest species
Antarctic	0.99	0.001	ASV456	Actinomycetota	Microbacteriaceae	Microbacteriaceae
	0.96	0.001	ASV4	Bacteroidota	Flavobacterium	Flavobacterium_unclassified
	0.91	0.001	ASV33	Patescibacteriota	Candidatus_Nomurabacteria	uncultured_bacterium
	0.91	0.001	ASV54	Patescibacteriota	Berkelbacteria	uncultured_bacterium
	0.91	0.001	ASV55	Patescibacteriota	Candidatus_Nomurabacteria	Candidatus_Nomurabacteria
	0.82	0.001	ASV103	Patescibacteriota	Parcubacteria_unclassified	Parcubacteria_unclassified
	0.82	0.001	ASV14	Patescibacteriota	Parcubacteria	uncultured_bacterium
	0.82	0.001	ASV89	Patescibacteriota	Candidatus_Kaiserbacteria	metagenome
	0.81	0.001	ASV279	Patescibacteriota	Saccharimonadales	Saccharimonadales_unclassified
	0.82	0.001	ASV541	Pseudomonadota	Burkholderia-Caballeronia-Paraburkholderia	Burkholderia-Caballeronia-Paraburkholderia
	0.82	0.001	ASV592	Pseudomonadota	Pelomonas	Pelomonas_saccharophila
	0.82	0.001	ASV757	Pseudomonadota	Ralstonia	Ralstonia_unclassified
	0.80	0.001	ASV778	Pseudomonadota	Polaromonas	Polaromonas_unclassified
	0.91	0.001	ASV13	Bacillota	Desulfosporosinus	Desulfosporosinus_unclassified
	0.82	0.001	ASV150	Bacillota	Clostridium_sensu_stricto_13	Clostridium_estertheticum
Non Antarctic	0.93	0.001	ASV155	Actinomycetota	hgcI_clade	Candidatus_Nanopelagicus
	0.99	0.001	ASV837	Bacteroidota	Fluviicola	Fluviicola_unclassified
	0.89	0.001	ASV264	Bacteroidota	Sediminibacterium	Sediminibacterium_unclassified
	0.86	0.001	ASV86	Pseudomonadota	Comamonadaceae_unclassified	Comamonadaceae_unclassified

## Discussion

Our study demonstrates that microbial communities in Antarctic lakes differ markedly from those in non-polar oligotrophic lakes, reflecting adaptations to the extreme Antarctic environment. Even within King George Island, lake-specific environmental gradients shaped contrasting taxonomic and ecological assemblages. Moreover, the prevalence of hybrid ASVs indicates that both environmental filtering and dispersal jointly influence community structure. These findings show that Antarctic lake microbiota are not only highly sensitive to local environmental change but also play active roles in ecosystem processes.

### Distinct microbial diversity and adaptations in Antarctic lakes compared with global freshwater ecosystems

Microbial communities in Antarctic lakes exhibit significant differences from those in temperate lakes on other continents. In general, in freshwater ecosystems, microbial communities are dominated by Pseudomonadota, Actinomycetota, and Cyanobacteriota, which play significant roles in primary production and carbon cycling^45^. However, Antarctic lakes exhibited different microbial communities dominated by Bacteroidota, Actinomycetota, and Pseudomonadota. The microbial compositions in Antarctic lakes could result from harsh environmental conditions, including low temperatures, prolonged darkness, strong UV radiation during summer, and limited nutrient availability^3–5^. Specifically, the dominance of Bacteroidota across our samples could indicate their essential role in degrading complex organic materials prevalent in Antarctic freshwater ecosystems, where the input of allochthonous organic material from surrounding environments influences microbial community structure^46,47^.

Our ISA results revealed *Flavobacterium* (phylum Bacteroidota) as an important indicator genus in several Antarctic lakes on King George Island. Members of *Flavobacterium* are well-known psychrotolerant bacteria that produce cold-active extracellular enzymes involved in degrading high-molecular-weight organic compounds such as polysaccharides, proteins, and lipids under cold conditions^48^. Furthermore, we detected *Polaromonas* and *Pelomonas* as indicating taxa in Antarctic freshwater ecosystems. *Polaromonas*, in particular, is frequently isolated from polar regions due to its psychrophilic adaptations and metabolic versatility, such as aerobic chemoheterotrophy and facultative hydrogen oxidation, which support its survival under nutrient-poor and extreme conditions^49^. Notably, Patescibacteriota, identified as an indicator group in Antarctic lakes, are highly adapted to oligotrophic and extreme environments due to their reduced genome size (~ 1 Mbp) and cellular dimensions (~ 0.3 μm), allowing efficient nutrient uptake even under extreme nutrient limitation^50^. Their significant abundance highlights their critical ecological role as oligotrophic specialists uniquely suited to the Antarctic freshwater niche.

Overall, the marked differences in microbial composition between Antarctic and non-Antarctic freshwater ecosystems reflect adaptive specialization driven by the extreme and oligotrophic Antarctic environment, which favors taxa with unique metabolic capabilities to cope with persistent environmental stressors and resource scarcity^51,52^. Our findings also have broader implications for understanding ecosystem responses to ongoing climate change. The microbial communities observed in Antarctic lakes suggest that these systems may be highly sensitive to environmental disturbances. Such sensitivity could render them vulnerable to abrupt ecological shifts if thresholds in salinity, nutrient inputs, or temperature are crossed. Microbial communities, therefore, can act as early indicators of regime shifts, providing valuable insight into ecosystem resilience under changing polar conditions. Recognizing these microbial responses is crucial for anticipating ecological tipping points and for developing strategies to manage and protect fragile Antarctic freshwater ecosystems in a warming world.

### Resource acquisition strategies and contributions to biogeochemical cycling in Antarctic lake microbial communities

Microbial communities in Antarctic lakes, including the water column and sediments, play central roles in biogeochemical cycling and adapt to severely limited nutrient availability through diverse metabolic strategies. The high abundances of taxa such as Bacteroidota and Verrucomicrobiota in lake sediments suggests their active participation in organic matter decomposition and carbon cycling^53^. The sediments analyzed in this study presented relatively high TOC, TN, and water content values, especially in LB and LS, indicating the presence of active microbial decomposition processes facilitated by sediment-associated microbial communities. The high abundances of Bacteroidota, particularly members of the genus *Flavobacterium*, likely contribute significantly to organic matter remineralization and subsequent nutrient recycling^48^. Consistent with these taxonomic patterns, the predictive potential for Glycolysis via EMP pathway was significantly higher in LB and LS sediments, supporting the role of these communities in driving organic matter degradation.

Clear differences were observed in the ways nitrogen is acquired among the Antarctic lakes (Fig. 4). Overall, the potential for nitrogen fixation was very low, but sediments from LB showed relatively higher levels, suggesting some capacity to convert atmospheric nitrogen to ammonium. By contrast, water samples appeared to rely more on mineralization and assimilatory nitrate reduction. These pathways are energetically less demanding than nitrogen fixation and may represent an adaptive strategy for microbial growth under oligotrophic and nutrient-limited conditions. Compared to water samples, sediments generally showed lower potentials for mineralization and assimilatory nitrate reduction. In LP sediments, the potential for *nir*BD was higher, suggesting a stronger role for DNRA. In contrast, sediments from LB and LS showed elevated potentials for *nrf*AH, which is associated with DNRA under strongly reducing conditions. The dominance of *nrf*AH in LB and LS likely reflects their geochemical conditions, including higher organic carbon and sulfate availability, which create anoxic environments favorable for this process. These results show that DNRA is a key nitrogen pathway in Antarctic lake sediments, but the specific genes involved vary across lakes depending on their redox conditions and nutrient availability.

Differences were also observed in sulfur utilization among the lakes. In water samples, sulfur was primarily acquired through assimilatory sulfate reduction, while both water and sediment samples from LB and LS showed elevated potential for sulfur mineralization. Notably, sulfate-reducing taxa (e.g., Desulfocapsaceae and Desulfobacterota) and sulfur-oxidizing genera (e.g., *Sulfurimonas* and *Thiobacillus*) were prevalent in LS sediments, where sulfate concentrations were exceptionally high (> 700 ppm). The co-occurrence of sulfate reducers and oxidizers suggests active sulfur cycling in the sediment environment of LS, indicating tightly coupled sulfur transformation that may be supported by marine-derived sulfate inputs and anoxic sediment conditions. Such sulfur-cycling microbial communities affect sediment geochemistry, potentially impacting the broader aquatic ecosystem by modulating the availability of redox-sensitive nutrients^9,54^.

The concentrations of methane and nitrous oxide, which were also significant environmental variables identified by the envfit analysis, suggest that microbial communities play active roles in carbon and nitrogen cycling in these lakes. Although the predictive potential for methane oxidation and methanogenesis was generally low, the detection of methylotrophic taxa such as Methylomirabilota in sediments suggests ongoing microbial methane oxidation that may reduce methane release to the atmosphere^55^. In addition, the LS water samples, which showed elevated nitrous oxide concentrations, also exhibited high potential for *nor*B, a gene encoding nitric oxide reductase involved in the conversion of nitric oxide to nitrous oxide. These findings highlight the important role of Antarctic lake microbial communities in shaping environmental conditions and suggest that they may help regulate greenhouse gas emissions through both anaerobic and aerobic oxidation processes.

### Environmental determinants influencing microbial differentiation among Antarctic lakes

Although geographically proximal, the Antarctic lakes exhibited considerable differences in microbial community composition, driven primarily by specific environmental conditions. Environmental factors such as salinity, sulfate, methane concentrations, elevation, and distance to the shoreline were significantly correlated with microbial community differentiation among the lakes, as confirmed by our NMDS and envfit analyses (Fig. 3(b)).

LS, characterized by brackish conditions and exceptionally high salinity and sulfate concentrations, presented the unique microbial community structure. While nutrient levels varied among the lakes, our results indicate that salinity showed the strongest influence on community differentiation. Marine-derived taxa such as Polaribacter and sulfur-cycling groups (*Sulfurimonas* and Desulfocapsaceae) were particularly abundant in LS, suggesting that salinity-driven selective pressures, rather than nutrient availability alone, were key in shaping its unique microbial assemblage. This pattern also indicates the close proximity of LS to the ocean and substantial marine influences, further reinforcing the dominant role of salinity in structuring its microbial community^56–58^. Environmental filtering is thus apparent, with lake-specific conditions, such as the high salinity in LS or the strong redox gradients in sediments, selecting for specialized taxa confined to particular habitats.

Conversely, the microbial communities of lakes LF and LP, with comparatively lower conductivity and nutrient concentrations, clustered closely together, suggesting environmental similarity. However, LF was unique in its particularly high proportion of water-specific ASVs, which may reflect limited terrestrial or sediment inputs due to the lake’s greater depth (15 m) that facilitate the dominance of truly aquatic-adapted microorganisms. This pattern aligns with previous studies identifying lake depth as a key environmental determinant of microbial composition due to its influence on physical mixing, sediment interactions, and nutrient availability^59,60^.

The significant elevation of LW appears to select for psychrophilic taxa, such as *Cryobacterium*, which is adapted specifically to the cold, harsh glacial conditions prevalent at higher elevations^61^. Such elevation-dependent microbial differentiation suggests that even small-scale variations in topographic position could influence microbial assembly processes in Antarctic lake ecosystems.

Moreover, our data revealed pronounced sediment-depth differentiation in microbial communities, suggesting distinct environmental gradients within sediment layers (e.g., redox potential, nutrient availability, and organic matter type). Upper sediment layers exhibited microbial communities that were similar to those in water columns, indicating continuous microbial exchange at the sediment-water interface. Deeper sediments, however, diverged substantially, likely due to reduced oxygen availability and increasingly anaerobic conditions, which resulted in selection for anaerobic taxa capable of fermentative metabolism, sulfate reduction, or methanogenesis^62,63^.

While environmental filtering clearly shaped lake-specific microbial assemblages through factors such as salinity, sulfate concentration, and redox gradients, evidence from ASV distributions points to the additional role of dispersal processes. The high proportion of hybrid ASVs observed across lakes, together with the dominance of core ASVs in surface water samples exposed to the atmosphere, suggests that dispersal between habitats contributes substantially to microbial assembly. It is further supported by the high abundance of hybrid ASVs at the water-sediment interface, consistent with continuous exchange of microorganisms between these environments. Functionally, the detection of anaerobic pathways such as denitrification and DNRA in the oxygenated water column also indicates that microbial taxa can be dispersed from sediments and persist transiently under non-optimal conditions. These findings suggest that Antarctic lake microbial communities are structured by both strong environmental filtering and frequent dispersal across connected habitats, with their relative importance varying depending on local environmental gradients.

In summary, the lakes displayed distinct microbial community compositions compared with those of freshwater ecosystems on other continents, with notable dominance of Bacteroidota, Actinomycetota, and Pseudomonadota. These differences highlight unique microbial adaptations to the extreme and oligotrophic conditions prevalent in Antarctic lake environments. The environmental drivers, such as salinity and sulfate concentration, shaped microbial community differentiation among the lakes, especially in brackish, sulfate-rich LS. Sediment communities were significantly influenced by water content, TOC, TN, and biogenic silica content, emphasizing the importance of sediment geochemistry. The identified critical environmental gradients not only differentiate microbial assemblages across habitats but also influence their contributions to biogeochemical cycling. In addition, Antarctic lake microbial communities are shaped by both environmental filtering and dispersal, with these processes together influencing biodiversity and ecosystem functions under extreme conditions. These findings emphasize the importance of local environmental conditions in determining Antarctic microbial biodiversity and ecological functions, warranting further studies focusing on microbial adaptations and ecosystem-scale effects under ongoing climate change.

## Supplementary Information

Below is the link to the electronic supplementary material.

Supplementary Material 1

## Data Availability

All raw sequence data have been submitted to the Korea Polar Data Center under the study accession numbers KOPRI-KPDC-00002604 (https://dx.doi.org/doi:10.22663/KOPRI-KPDC-00002604).
