# Supplementary material for: Environmental gradients shape microbial community structure and ecosystem processes in Antarctic lakes on King George Island
Source: Sci Rep. 2025 Oct 27;15:37519. doi: 10.1038/s41598-025-21587-1 (PMC12559251; doi:10.1038/s41598-025-21587-1)
Supplement: Supplementary file 1 — Supplementary Material 1 [file 41598_2025_21587_MOESM1_ESM.docx]

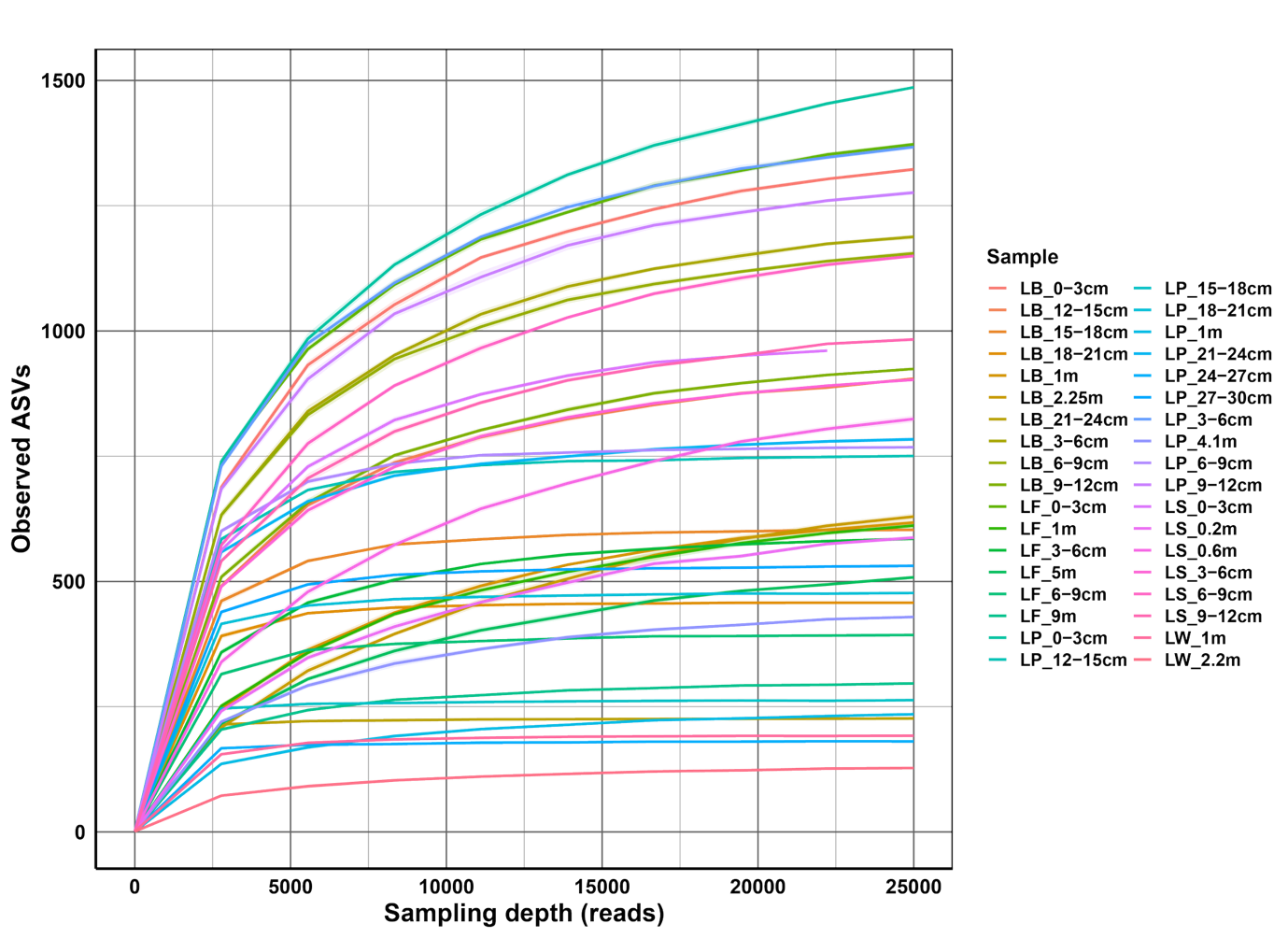


**Fig. S1. Rarefaction curves based on amplicon sequencing of microbial communities from water and sediment samples collected in Antarctic lakes.** Each curve represents the relationship between sequencing depth (subsampled reads) and the observed number of amplicon sequence variants (ASVs) for individual samples. Curves were generated using rarefaction with 10 random subsamplings per depth, and the mean values are shown with shaded areas indicating the 95% confidence interval.

**Fig. S2. Microbial composition of water and sediment samples from five Antarctic lakes.** The top 10 major phyla of the entire community are shown.

**Table S1.** Sources and sequencing details of reference freshwater lake datasets included for global comparison

| **Lake name** | **Country** | **Sampling Depth** | **Sequencer** | **Regions** | **SRA ID** | **References** |
| --- | --- | --- | --- | --- | --- | --- |
| Flathead | USA | 5-90 m | IlluminaMiSeq | V3-V4 | SRR5318985  SRR5318986  SRR5318991  SRR5318992  SRR5318997  SRR5318998 | https://doi.org/10.1002/lno.12509 |
| Pavin | Europe | 0.5-30m | IlluminaMiSeq | V3–V4 | SRR10165128  SRR10165129  SRR10165130  SRR10165131 | <http://dx.doi.org/10.1007/s00248-018-1143-y> |
| Labynkyr | Russia | 1-25m | IlluminaMiSeq | V3-V4 | SRR24598503  SRR24598504  SRR24598505  SRR24598506  SRR24598507  SRR24598508  SRR24598509  SRR24598510  SRR24598511  SRR24598512  SRR24598513  SRR24598514  SRR24598515  SRR24598516  SRR24598517  SRR24598518  SRR24598519  SRR24598520  SRR24598521 | <http://dx.doi.org/10.1007/s00248-021-01912-7> |
